# Increasing Incidence of Invasive Group A *Streptococcus* Disease in First Nations Population, Alberta, Canada, 2003–2017

**DOI:** 10.3201/eid2702.201945

**Published:** 2021-02

**Authors:** Gregory J. Tyrrell, Christopher Bell, Lea Bill, Sumana Fathima

**Affiliations:** University of Alberta, Edmonton, Alberta, Canada (G.J. Tyrrell);; Alberta Precision Laboratories–Public Health–Alberta Health Services, Edmonton (G.J. Tyrrell);; Alberta Ministry of Health, Edmonton (C. Bell, S. Fathima);; Alberta First Nations Information Governance Center, Siksika, Alberta, Canada (L. Bill)

**Keywords:** First Nations, invasive group A streptococcus disease, iGAS, *Streptococcus pyogenes*, *emm* type, high incidence, Alberta, Canada, GAS, bacteria, streptococci

## Abstract

The incidence of invasive group A *Streptococcus* (iGAS) disease in the general population in Alberta, Canada, has been steadily increasing. To determine whether rates for specific populations such as First Nations are also increasing, we investigated iGAS cases among First Nations persons in Alberta during 2003–2017. We identified cases by isolating GAS from a sterile site and performing *emm* typing. We collected demographic, social, behavioral, and clinical data for patients. During the study period, 669 cases of iGAS in First Nations persons were reported. Incidence increased from 10.0 cases/100,000 persons in 2003 to 52.2 cases/100,000 persons in 2017. The 2017 rate was 6 times higher for the First Nations population than for non–First Nations populations (8.7 cases/100,000 persons). The 5 most common *emm* types from First Nations patients were 59, 101, 82, 41, and 11. These data indicate that iGAS is severely affecting the First Nations population in Alberta, Canada.

GAS disease is caused by the gram-positive coccus bacterium *Streptococcus pyogenes*; invasive GAS (iGAS) disease is typically defined as identification of GAS from any sterile site, including blood, cerebrospinal fluid, brain, and deep tissues. GAS affects persons worldwide and causes a wide array of diseases including pharyngitis, skin infections (e.g., impetigo and cellulitis), bacteremia, pneumonia, septic arthritis, rheumatic fever, rheumatic heart disease, and the severe invasive diseases necrotizing fasciitis and streptococcal toxic shock syndrome (*1*,*2*). The epidemiology of many of these diseases varies by region; pharyngitis is more common in high-income countries, and diseases such as impetigo are more common in tropical climates and low-income countries (*3*,*4*). In 2005, the mortality rate associated with GAS disease (noninvasive and invasive) was ≈500,000 deaths/year (*2*).

GAS bacteria can be typed by identifying variability in the DNA sequence at the tip of a coiled-coil protein on the bacteria’s surface (the M protein), which is encoded by the *emm* gene. Worldwide, there are >240 *emm* types (*5*,*6*). Prevalence of *emm* types varies according to population and geography (*7*). In addition, the diversity of *emm* types is greater in developing countries and less in more developed countries (*8*–*10*).

Previous studies have shown that rates of iGAS disease are higher for indigenous populations than for other populations (*11*–*15*). Examples include Native Americans in Arizona and Alaska and indigenous communities in parts of Australia and northwestern Ontario, Canada. For parts of the country such as western Canada, detailed descriptive data on iGAS in the indigenous population are lacking. We previously reported increased age-standardized rates of iGAS in Alberta’s general population and increasing incidence from a low of 4.2 cases/100,000 persons in 2003 to a high of 10.2 cases/100,000 persons in 2017 (*16*). On the basis of that finding, we explored whether iGAS rates also increased for the First Nations population of Alberta during the same period.

## Methods

### Case and Population Data

All iGAS cases were identified by diagnostic microbiology laboratories in Alberta, where iGAS disease is listed as a Public Health Notifiable Disease (https://open.alberta.ca/publications/streptococcal-disease-group-a-invasive). All cases identified by diagnostic microbiology laboratories are required to be reported to the Alberta Ministry of Health. Confirmed iGAS cases are defined as identification of GAS from any typically sterile site, including blood, cerebrospinal fluid, brain, deep tissues, and joints (https://open.alberta.ca/publications/streptococcal-disease-group-a-invasive). After initially identifying iGAS isolates, diagnostic microbiology laboratories in Alberta informed provincial public health officials, and trained public health nurses collected clinical and risk factor data according to routine notifiable disease requirements by using a notifiable disease reporting form (https://open.alberta.ca/publications/ndr-manual-9th-edition). Clinical (including risk factors) and laboratory data were electronically captured in the Alberta Health Communicable Disease Reporting System (CDRS), an electronic database held by Alberta Health and used to capture data regarding cases of reported communicable disease. Staff at Alberta Health reviewed each incident case for data quality and completeness in the CDRS.

For the risk factor analysis, we defined addiction abuse as a primary chronic neurobiological disease with genetic, psychosocial, and environmental factors and behaviors leading to impaired control over drug use, compulsive use, continued use despite harm, and craving. Subsets of addiction abuse were alcohol abuse and drug use. Alcohol abuse was defined as the overindulgence in alcohol, leading to effects that are detrimental to the person’s physical and mental health. Drug use was defined as the use of all drugs that were acquired unlawfully. Deaths were determined at the time of data collection by Alberta Health.

In Canada, there are 3 groups of aboriginal peoples: First Nations, Inuit, and Métis (https://www.rcaanc-cirnac.gc.ca/eng/1100100013785/1529102490303). Only cases in First Nations persons, Inuit, and Métis were captured in this analysis. To identify cases in First Nations persons only, we extracted all iGAS cases during 2003–2017 from the CDRS and used a Unique Lifetime Identifier number to link them to the Alberta Health First Nations identifiers registry held by Alberta Health. The First Nations registry includes anyone ever registered as having First Nations status. For statistical analyses, we used deidentified and aggregated data. The First Nations population of Alberta in 2003 was 140,436; in 2017, the population was 164,786 (http://www.ahw.gov.ab.ca/IHDA_Retrieval). An ethical framework for information and knowledge-sharing for this project was provided by the principles of OCAP (Ownership, Control, Access and Possession) within Alberta First Nations (http://afnigc.ca/main/index.php?id=resources&content=community%20resources).

### *emm* Typing of iGAS Isolates

All GAS isolates from persons with invasive cases are required to be submitted to the Provincial Public Health Laboratory for *emm* typing. The method used to type iGAS isolates from 2003 through September 2006 was a previously described serologic typing assay (*17*). From October 2006 through 2017, *emm* typing was conducted by DNA sequencing of the M serotype specific region of the *emm* gene as previously described (*17*–*19*). Assignment of *emm-*cluster type was performed as previously described (*20*). In brief, after the *emm* type was identified, it was matched to an *emm*-cluster type on the basis of the typing scheme of Sanderson-Smith et al. (*20*).

### Statistical Analyses

During 2003–2017, First Nations population estimates in Alberta were extracted from the online Interactive Health Data Application database (http://www.ahw.gov.ab.ca/IHDA_Retrieval). We calculated incidence rates by age group and by year of diagnosis, expressed as cases per 100,000 persons. Data were analyzed by using SAS version 9.3 (SAS Institute Inc., https://www.sas.com) and graphed by using OriginLab software 2018 (OriginLab Corporation, https://www.originlab.com). To compare clinical presentations and *emm* clusters between First Nations and non–First Nations persons, we conducted Fisher exact *t* tests. We considered p<0.05 to be statistically significant.

## Results

### Incidence

**Figure 1:**
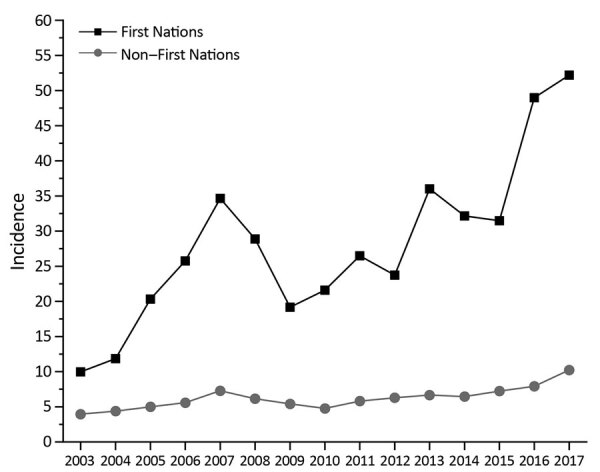
Incidence (cases/100,000 population) of invasive group A *Streptococcus* disease for First Nations and non–-First Nations populations, Alberta, Canada, 2003–2017. The incidence rate for the First Nations population climbed from a low of 10.0 in 2003 to a high of 52.2 in 2017. This rate contrasts with that for the non–First Nations population (3.7 in 2003 and 8.7 in 2017).

**Figure 2:**
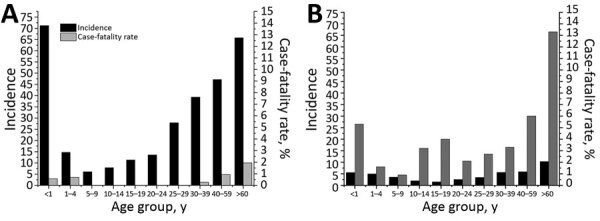
Incidence (cases/100,000 population) and case-fatality rates for invasive group A *Streptococcus* disease for First Nations (A) and non–First Nations (B) populations, by age group, Alberta, Canada, 2003–2017.

**Figure 3:**
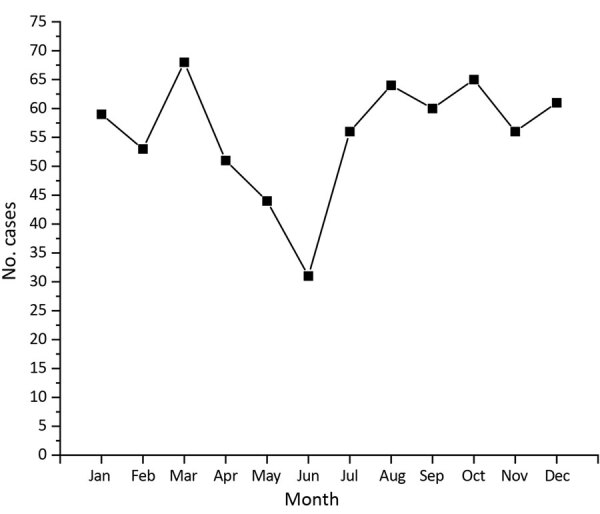
Seasonality of invasive group A *Streptococcus* disease in the First Nations population, Alberta, Canada, 2003–2017.

Over the 15 years reviewed, we found 669 cases of iGAS in the First Nations population in Alberta; mean annual incidence rate was 28.6 cases/100,000 persons. The number of cases in 2003 was 14, which by 2017 increased to 86. In 2017, the incidence rate for the Alberta First Nations population (52.2 cases/100,000 persons) was 6 times greater than that for non–First Nations populations (8.7 cases/100,000 persons) (Figure 1). By First Nations age group, incidence was highest among persons <1 year of age (71.2 cases/100,000 persons), followed by persons >60 years of age (65.8 cases/100,000 persons) (Figure 2, panel A). iGAS incidence among First Nations persons of all age groups was higher than that among non–First Nations persons (Figure 2). Incidence rates varied by season; the number of cases of iGAS among First Nations persons was lowest during May and June (Figure 3), similar to what has been reported for the general population (*16*).

### Case Demographics, Clinical Manifestations, and Risk Factor Analyses

The median age of First Nations persons with iGAS disease was 38.5 years, younger than the overall median age of 45 years for persons with iGAS disease previously reported for the overall Alberta population (*16*). The proportion of First Nations iGAS patients who were male (54.8%) was similar to the proportion of non–First Nations patients who were male (58.5%). A total of 24 deaths among First Nations patients were attributed to iGAS; case-fatality rate was 3.6%. In comparison, the case-fatality rate among non–First Nations persons was 7.0%. By age group, of the 24 First Nations persons who died, 2 were children (<1 through 2 years of age). The remaining 22 First Nations persons who died were >35 years of age (Figure 2, panel A). For all age groups, case-fatality rates were higher among non–First Nations than among First Nations persons (Figure 2, panels A and B).

We observed little difference between First Nations and non–First Nations populations with respect to clinical diagnosis (Table 1). The percentage of soft tissue infections was higher for the First Nations population than the non–First Nations population (18.8% vs. 10.8%, p<0.001; Table 1). Frequency of streptococcal toxic shock syndrome was greater in the non–First Nations population than in the First Nations population (6.4% vs. 2.3%, p<0.001; Table 1). The most prevalent risk factors for the First Nations population over the 15-year study period were addiction abuse, alcohol abuse, drug use, nonsurgical wounds, homelessness, diabetes mellitus, and hepatitis C (*16*) (Table 2).

**Table 1:** Invasive group A *Streptococcus* disease in First Nations and non–First Nations persons, by clinical diagnosis, Alberta, Canada, 2003‑2017*

System, clinical condition	First Nations, no. (%) cases	Non–First Nations, no. (%) cases	p value†
Blood, brain, sterile tissue			
Septicemia/bacteremia	319 (37.8)	1570 (42.8)	0.011
Streptococcal toxic shock syndrome	19 (2.3)	235 (6.4)	<0.001
Meningitis	9 (1.1)	16 (0.4)	0.061
Peritonitis	5 (0.6)	24 (0.7)	0.886
Encephalitis	1 (0.1)	0	0.373
Skin/soft tissue			
Cellulitis	146 (17.3)	633 (17.3)	0.971
Soft tissue infection	159 (18.8)	397 (10.8)	<0.001
Necrotizing fasciitis	60 (7.1)	266 (7.3)	0.989
Respiratory			
Pneumonia	50 (5.9)	291 (7.9)	0.054
Epiglottitis	2 (0.2)	11 (0.3)	0.824
Bone			
Joint	63 (7.5)	192 (5.2)	0.016
Osteomyelitis	10 (1.2)	27 (0.8)	0.272
Unknown	2 (0.2)	0	0.069
Total	845 (100)	3,688 (100)	Not applicable

*Patients may have multiple clinical manifestations (193 patients had 2 clinical manifestations, 39 patients had 3, 2 patients had 4, and 1 had 5). †By Fisher exact test.

**Table 2:** Risk factors for First Nations and non–First Nations persons with invasive group A *Streptococcus* disease, Alberta, Canada, 2003‑2017*

Risk factor	No. (%)	
	First Nations, n = 669	Non–First Nations, n = 2,315
Diabetes	103 (15.4)	176 (7.6)
Hepatitis C	101 (15.1)	181 (7.8)
Immunocompromised	41 (6.1)	238 (10.3)
Nonsurgical wound	165 (24.7)	543 (23.5)
Surgical wound	43 (6.4)	133 (5.7)
Addiction abuse	250 (37.4)	390 (16.8)
Alcohol abuse	188 (28.1)	90 (3.9)
Drug use	126 (18.8)	307 (13.3)
Homelessness	117 (17.5)	257 (11.1)

*Percentages may add up to >100% because each patient may have multiple risk factors.

### *emm* Types and *emm* Cluster Descriptions

**Figure 4:**
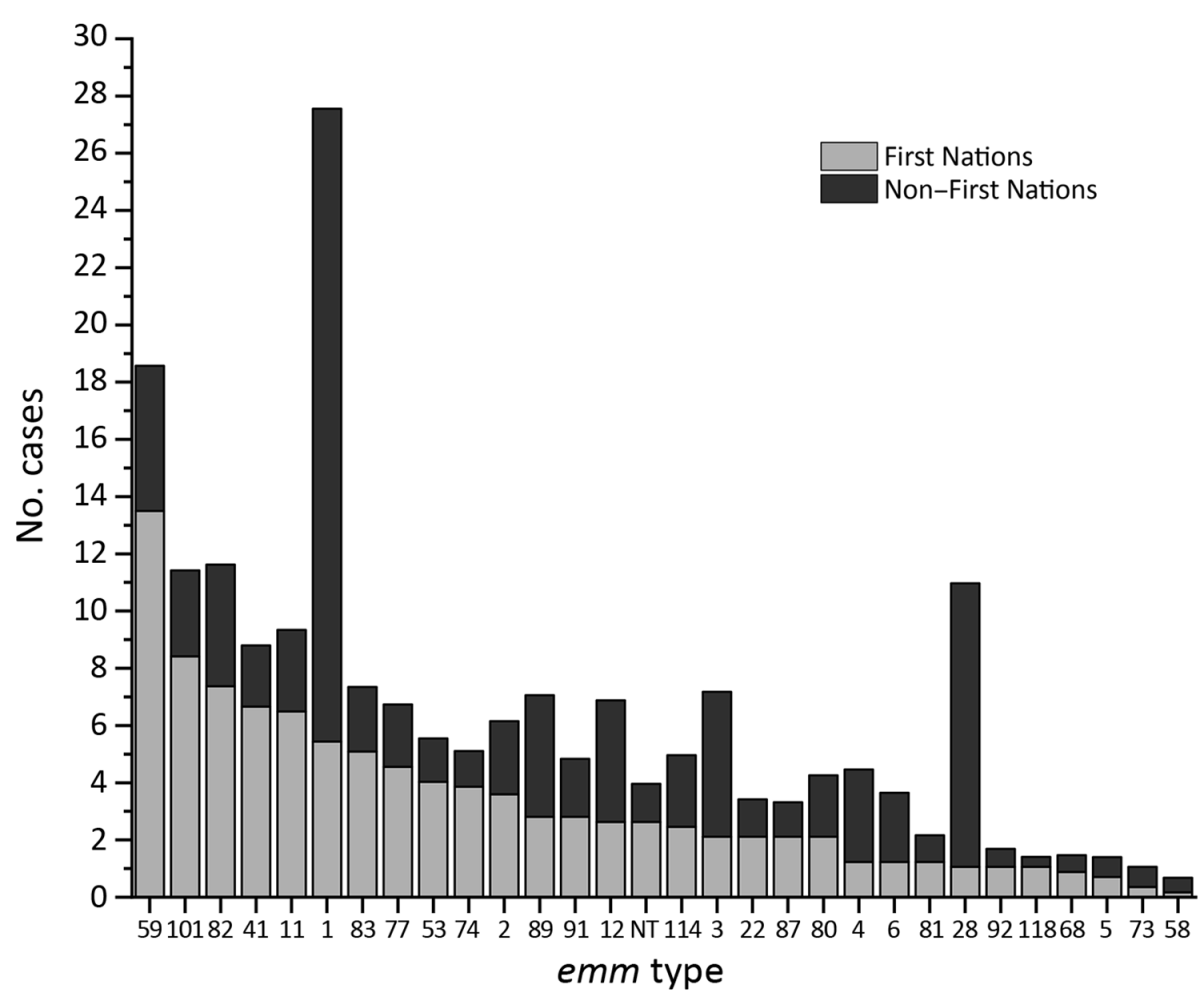
Group A *Streptococcus* *emm* types from First Nations persons and non–First Nations with invasive disease, Alberta, Canada, 2003–2017.

For the 15-year study period, we observed a difference in the distribution of *emm* types between First Nations and non–First Nations populations in Alberta. The most prevalent *emm* type among the First Nations population was *emm*59, which accounted for 13.5% of all *emm* types, followed by *emm*101 (8.4%) and 82 (7.4%) (Table 3, Figure 4). This finding was in contrast to that for the non–First Nations population, for which the top 3 *emm* types were *emm*1 (22.1%), 28 (9.9%), 3 (5.1%), and 59 (5.1%).

**Table 3:** Number of *emm* gene types in group A *Streptococcus* from First Nations persons with invasive disease, by year, Alberta, Canada, 2003–2017*

*emm* type	2003	2004	2005	2006	2007	2008	2009	2010	2011	2012	2013	2014	2015	2016	2017	Total
59	0	0	1	0	7	18	12	4	3	1	3	1	4	10	13	77
101	0	0	0	0	0	1	4	1	4	2	1	2	9	14	10	48
82	1	3	0	3	7	2	1	0	2	3	6	2	2	7	3	42
41	1	1	4	2	2	1	0	0	0	3	3	11	4	4	2	38
11	0	0	0	2	0	0	0	0	2	5	3	0	5	9	11	37
1	0	1	1	1	4	2	1	2	3	1	5	5	1	0	4	31
83	0	1	2	2	6	2	0	0	1	3	1	1	2	3	5	29
77	0	1	0	1	0	0	1	2	2	5	11	2	0	0	1	26
53	0	0	0	2	2	1	2	2	5	1	5	3	0	0	0	23
74	0	0	0	0	0	0	0	0	0	0	0	0	0	5	17	22
89	0	0	2	1	2	0	1	2	4	0	1	1	1	0	1	16
91	0	0	0	3	1	0	0	0	1	2	3	1	3	2	0	16
12	0	0	1	1	1	0	0	2	4	0	1	0	1	3	1	15
114	0	2	1	2	3	0	0	2	1	0	0	2	1	0	0	14
3	1	0	0	1	1	0	0	0	1	0	0	0	5	3	0	12
22	1	0	0	0	0	0	0	2	0	0	2	3	1	2	1	12
87	0	0	0	1	1	0	0	2	3	0	1	2	1	1	0	12
80	0	0	1	0	0	4	0	2	1	1	2	0	0	1	0	12
Other	4	3	7	5	6	4	3	3	3	3	4	5	3	9	11	73
Nontypable	2	4	2	6	0	1	0	0	0	0	0	0	0	0	0	15
Total	10	16	22	33	43	36	25	26	40	30	52	41	43	73	80	570

*emm types found in >10 cases are shown.

*emm* cluster types differed substantially between First Nations and non–First Nations populations (Table 4). These differences were notable for cluster types A-C3, D4, E3, E4, and E6. The cluster types associated with the greatest number of cases for the First Nations population were D4 (*emm*41, 53, 80, 83, 91, 101) and E6 (*emm*11, 59, 75, 81, 94), representing 50.6% of the cases in this group. Twelve other clusters represented the remaining 49.4% (30 other *emm* types) of typed cases.

**Table 4:** *emm* clusters among group A *Streptococcus* from First Nations and non–First Nation persons with invasive disease, Alberta, Canada, 2003–2017

Cluster type	First Nations, no. (%)	Non–First Nations, no. (%)	Total cases	p value
A-C3	32 (5.8)	568 (22.7)	600	<0.001
A-C4	15 (2.7)	141 (5.6)	156	0.004
A-C5	12 (2.2)	130 (5.2)	142	0.001
D2	1 (0.2)	2 (0.1)	3	0.902
D3	0	3 (0.2)	3	0.948
D4	166 (30.0)	310 (12.4)	476	<0.001
E1	7 (1.3)	83 (3.1)	90	0.008
E2	18 (3.3)	64 (2.6)	82	0.435
E3	65 (11.7)	199 (8.0)	264	0.007
E4	79 (14.3)	616 (24.6)	695	<0.001
E5	0	5 (0.2)	5	0.736
E6	125 (22.6)	268 (10.7)	393	<0.001
M5	4 (0.7)	18 (0.7)	22	0.787
M6	7 (1.3)	62 (2.5)	69	0.100
M23	1 (0.2)	0	1	0.362
M74	22 (4.0)	32 (1.3)	54	<0.001
M111	0	1 (0.1)	1	0.408
M122	0	1 (0.1)	1	0.408
M218	0	1 (0.1)	1	0.408
Total	554 (100)	2,504 (100)	3,058	Not applicable

## Discussion

Our data illustrate the extent to which rates of iGAS disease are disproportionately higher for the First Nations population than the non–First Nations population in Alberta. For 2017, rates for the First Nations population (52.2 cases/100,000 persons) were 6-fold higher than rates for non–First Nations populations (8.7 cases/100,000 persons). Rates were also very high for First Nations children <1 year of age (71.2 cases/100,000 persons), in contrast to previously published rates for children in the 0 to 1–year age group of the general Alberta population (9.7 cases/100,000 persons [*16*]). Our results are similar to those reported for First Nations groups elsewhere. For example, another study in Canada found that, from 2009 through 2014, northwestern Ontario reported an elevated annualized rate of 56.2 cases/100,000 persons for the First Nations communities (*14*), similar to the rates we report for First Nations populations. With respect to other indigenous groups elsewhere, iGAS rates for the Aboriginal population in Australia during 2011–2013 were as high as 70.0 cases/100,000 persons, 8-fold higher than rates for the non-Aboriginal population (*21*). A previous study from Alaska found that during 2001–2013, the incidence rate for Alaska Natives was 13.7 cases/100,000 persons, compared with a rate of 3.9 cases/100,000 persons for non–Alaska Natives (*15*). Reported rates for Alaska Native children (39.9 cases/100,000 persons) have been higher than those reported for non–Alaska Native children (4.2 cases/100,000 persons) (*15*).

Drivers of the higher rates in the First Nations populations are not completely clear, although specific risk factors probably contribute. Risk factor data for iGAS in the First Nations population in our study frequently indicated nonsurgical wounds, addiction abuse (of which alcohol use and drug use are subsets), and homelessness. Other studies have noted high rates of GAS skin infections (e.g., cellulitis and abscesses) among persons who were experiencing homelessness and injected drugs (*22*–*24*). Recently, work by the Active Bacterial Core surveillance program in the United States showed that skin infections and skin breakdown were common among iGAS patients who were injection drug users or experiencing homelessness (*25*). These studies suggest that skin infections in vulnerable populations with these risk factors provide routes for iGAS infections.

A role of skin infections is also suggested when *emm* types are grouped by *emm* clusters. Grouping *emm* types by cluster shows that the bulk of disease among the First Nations population was focused on cluster *emm* types that are considered to be associated with skin-related infections (D clusters) and generalist strains (E clusters), as opposed to throat-related clusters (A–C) (*26*). This finding may suggest that in this population, skin-to-skin transmission occurs more frequently than respiratory route transmission. Opportunities for skin-to-skin transmission can include overcrowded households, as has been documented in Australia for the Aboriginal population, in whom the high burden of iGAS disease associated with skin and soft tissue infections is related to overcrowded or inadequate housing (*27*,*28*). With respect to other potential risk factors, risk for iGAS has been found to be significantly increased for close contacts of iGAS patients (≈2,000 times higher than background incidence) (*29*,*30*). Overcrowding and inadequate housing have also been documented among First Nations populations in Canada (*31*). Overcrowding has been considered endemic to First Nations populations in Canada and can probably lead to higher rates of disease than in non–First Nations populations (*31*). However, the numbers of persons living in households was not a demographic captured in this study; therefore, whether overcrowding was a contributor for this study remains unclear.

When we examined specific clinical conditions, we found additional contrasts in iGAS disease between First Nations and non–First Nations groups. Soft tissue and joint infections occurred with more statistically significant frequency in the First Nations population than in the non–First Nations population, whereas septicemia/bacteremia and streptococcal toxic shock syndrome occurred with more frequency in the non–First Nations population than in the First Nations population. The reasons for these differences are not clear and may be multifactorial. We did not expect to find that streptococcal toxic shock syndrome occurred more frequently in the non–First Nations population. A different *emm* type distribution may account for some of these differences.

Prevalence of *emm*1 was greater for the non–First Nations population (>22%) than for the First Nations population (<6%). *emm*1 is a major contributor to streptococcal toxic shock syndrome and is the most frequent *emm* type isolated from persons in the non–First Nations population in Alberta (*16*,*32*). The reason(s) behind the decreased presence of *emm*1 in the First Nations population despite it being the dominant *emm* type in the non–First Nations population are not clear.

In contrast to the lower frequency of streptococcal toxic shock syndrome is the higher frequency of soft tissue infections in the First Nations population. Our data show that *emm*59 was the most prevalent *emm* type in the First Nations population, and it has previously been shown that *emm*59 displays a tropism for skin infections (*33*,*34*). Since 2006, when a large outbreak of *emm*59 was first reported, *emm*59 has become an established *emm* type causing diseases such as skin and soft tissue infections throughout western Canada and the United States, whereas previously it was relatively rare (*33*,*35*–*37*). The *emm*59 cases reported here are probably derived from that original outbreak in 2006–2009 because before then, *emm*59 was uncommon.

Also notable is the striking difference in percentage of *emm*28 cases between First Nations (≈1%) versus non–First Nations (≈10%) populations. Our previous survey of the overall population indicated that *emm*28 was the second most common *emm* type after *emm*1 (*16*). *emm*28 falls within the E4 cluster categorizing this *emm* type as a generalist (*20*). The reason for the large difference in *emm*28 prevalence between the 2 populations is not clear.

The high iGAS incidence rate in the Alberta First Nations population illustrates the need for an effective GAS vaccine. One vaccine that has undergone phase 1 clinical trials is a polypeptide vaccine composed of 30 *emm* types (*38*). An assessment of the *emm* types contained in this 30-valent M protein–based GAS vaccine shows that this vaccine would include ≈53% of the *emm* types found in the Alberta First Nations population (*38*). If cross-protection against nonvaccine *emm* types based on immunogenicity in rabbits were included, this coverage rate would increase to 62.3% (*38*). In comparison, the 30-valent M-protein–based vaccine would include 77.1% of the *emm* types found in the non–First Nations population; if cross-protection with non-vaccine *emm* types were included, this percentage would increase to 79.8%. These comparisons do not include potential cross-protection through coverage of *emm* clusters. These *emm* type differences would have to be taken into account for the First Nations population should an *emm* type–based vaccine such as this be introduced into the Alberta population.

In summary, iGAS rates in the First Nations community in Alberta are high, at ≈50 cases/100,000 persons. Marked differences in iGAS disease in the First Nations population include more skin and soft tissue infections and fewer streptococcal toxic shock syndrome cases than in the non–First Nations population. Of note, substantial *emm* differences between the 2 populations could have potential implications for future vaccines.
